# The Discovery of an S-shaped Kidney in a Patient With Prostate Cancer: A Rare Finding

**DOI:** 10.7759/cureus.51685

**Published:** 2024-01-05

**Authors:** Stavros Tsiakaras, Georgios Langas, Vasileios Rafailidis, Dimitrios Memmos, Ioannis Mykoniatis, Irene Asouhidou, Paraskevi Karamitsou, Petros Sountoulides, Dimitrios Kikidakis, Ioannis Vakalopoulos, George K Paraskevas, Alexandros Poutoglidis

**Affiliations:** 1 1st Department of Urology, School of Medicine, Aristotle University of Thessaloniki, 'G. Gennimatas' General Hospital, Thessaloniki, GRC; 2 Clinical Radiology Department, School of Medicine, Aristotle University of Thessaloniki, AHEPA University Hospital of Thessaloniki, Thessaloniki, GRC; 3 Department of Anatomy and Surgical Anatomy, School of Medicine, Aristotle University of Thessaloniki, Thessaloniki, GRC; 4 Department of Otorhinolaryngology-Head and Neck Surgery, 'G. Papanikolaou' General Hospital, Thessaloniki, GRC

**Keywords:** s-shaped kidney, anatomical variations, crossed fused ectopic kidney, upper urinary tract, congenital abnormality

## Abstract

Crossed fused renal ectopia (CFRE) constitutes a rare congenital anomaly of the urinary tract, typically characterized by its predominantly asymptomatic nature and frequent incidental discovery. This case report delineates the clinical profile of a 56-year-old male admitted to our Prostate Cancer Outpatient Clinic due to elevated prostate-specific antigen (PSA) levels, ultimately leading to the diagnosis of prostate cancer. The patient was asymptomatic, with no family or surgical background. Notably, a fused ectopic kidney was incidentally identified during the staging process involving abdominal computed tomography (ACT) scanning. Remarkably, no additional abnormalities of the urinary tract or renal dysfunction manifested in this specific case. The significance of this report lies in the underscored emphasis on the importance of employing precise imaging techniques and tailored management strategies for patients harboring such anatomical variations.

## Introduction

Congenital abnormalities affecting the upper urinary tract encompass various disorders arising from embryonic defects [1, 2]. These anomalies can be attributed to various factors, including sporadic occurrences, genetic predisposition, and environmental influences. Their prevalence within the general population ranges from 3% to 11%, constituting approximately 50% of all congenital abnormalities [2]. These anomalies may impact various components such as the kidney, ureter, bladder, and urethra [3]. Crossed renal ectopia (CRE) represents a rare congenital anomaly wherein a kidney is situated on the contralateral side of its ureterovesical junction [1,3]. Most ectopic kidneys (90%) display fusion with their corresponding kidneys on the opposite side [1]. CRE is the second most prevalent form of renal fusion anomaly, after the horseshoe kidney [4]. The precise occurrence rate of CRE remains uncertain due to a significant proportion of patients remaining asymptomatic. In autopsy studies, the estimated prevalence is approximately 1 in 2000 cases [4, 5]. This condition demonstrates a higher prevalence in males, with a ratio of 3 to 2, and left-to-right crossover is more frequently observed with a ratio of 3 to 1 [4]. The diagnosis of renal fusion anomalies typically occurs in children and is frequently accompanied by other malformations. Additionally, it is identified in young adults during assessments for delayed menarche and in elderly individuals as incidental discoveries. The aim of our study is to emphasise the importance of employing precise imaging techniques and tailored management strategies for patients harboring such anatomical variations.

## Case presentation

A 56-year-old patient, with unremarkable medical history, was referred to our Prostate Cancer Outpatient Clinic due to the incidental discovery of elevated prostate-specific antigen (PSA) levels (PSA=119.7 ng/mL). Subsequently, the patient underwent a multiparametric magnetic resonance imaging (mpMRI), revealing a PI-RADS 5 lesion encompassing the entire prostate gland, notably affecting the posterior peripheral zone. The lesion demonstrated extension beyond the prostatic capsule, involving the neurovascular bundle. Furthermore, confirmation of pathological common iliac lymph nodes and potential bony metastases in the left femoral neck were observed.

**Figure 1:**
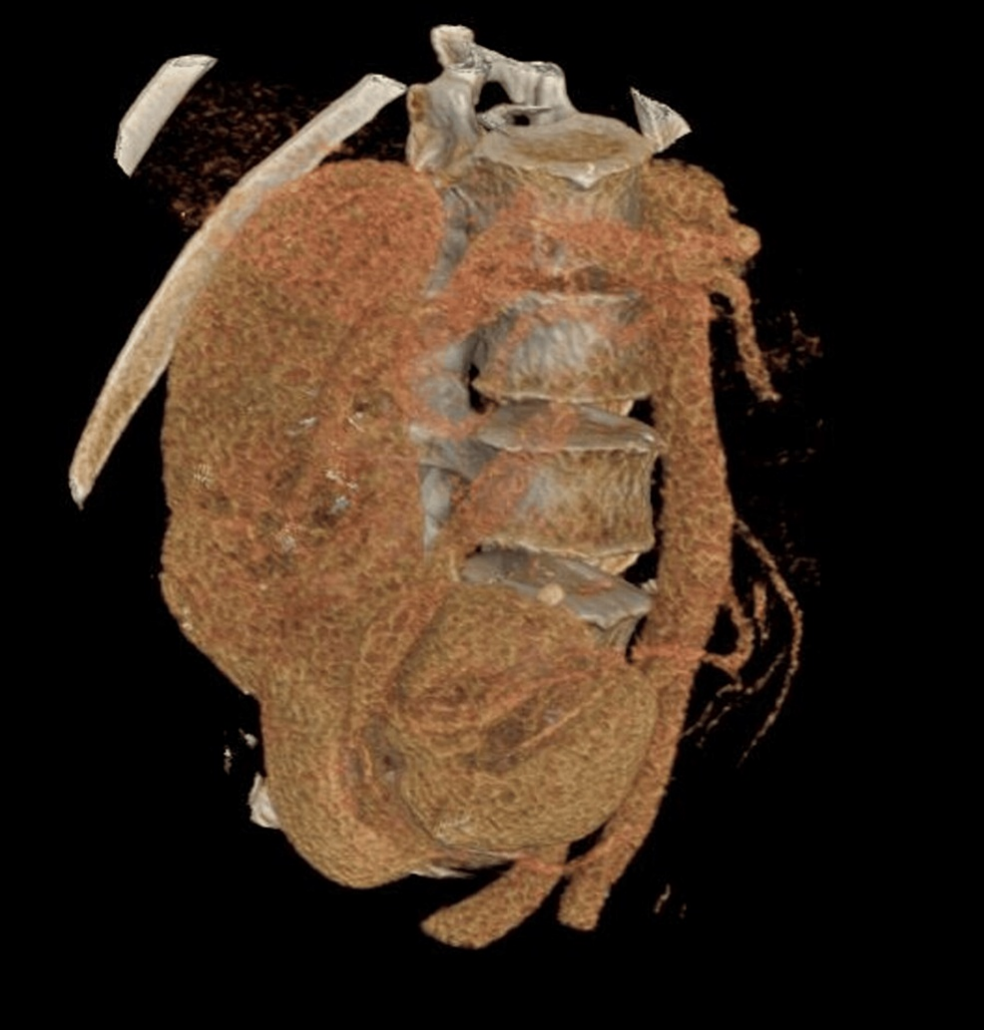
Volume rendering reformatted image of arterial phase computed tomography (CT) showing the S-shaped kidney. The kidney, its vasculature, and the lumbar spine were segmented based on density. The increased cranio-caudal length and the rotation of the kidney can be appreciated.

To verify the diagnosis, the patient underwent a transrectal ultrasound prostate biopsy, revealing a Gleason score of 5+4, score=9 prostate cancer. A Technitium-99m (Tc-99m) bone scan and a computed tomography (CT) of chest-abdomen-pelvis, lead to the ultimate staging of a de novo cT3aN1M1c prostate cancer (Figure 1). 

**Figure 2:**
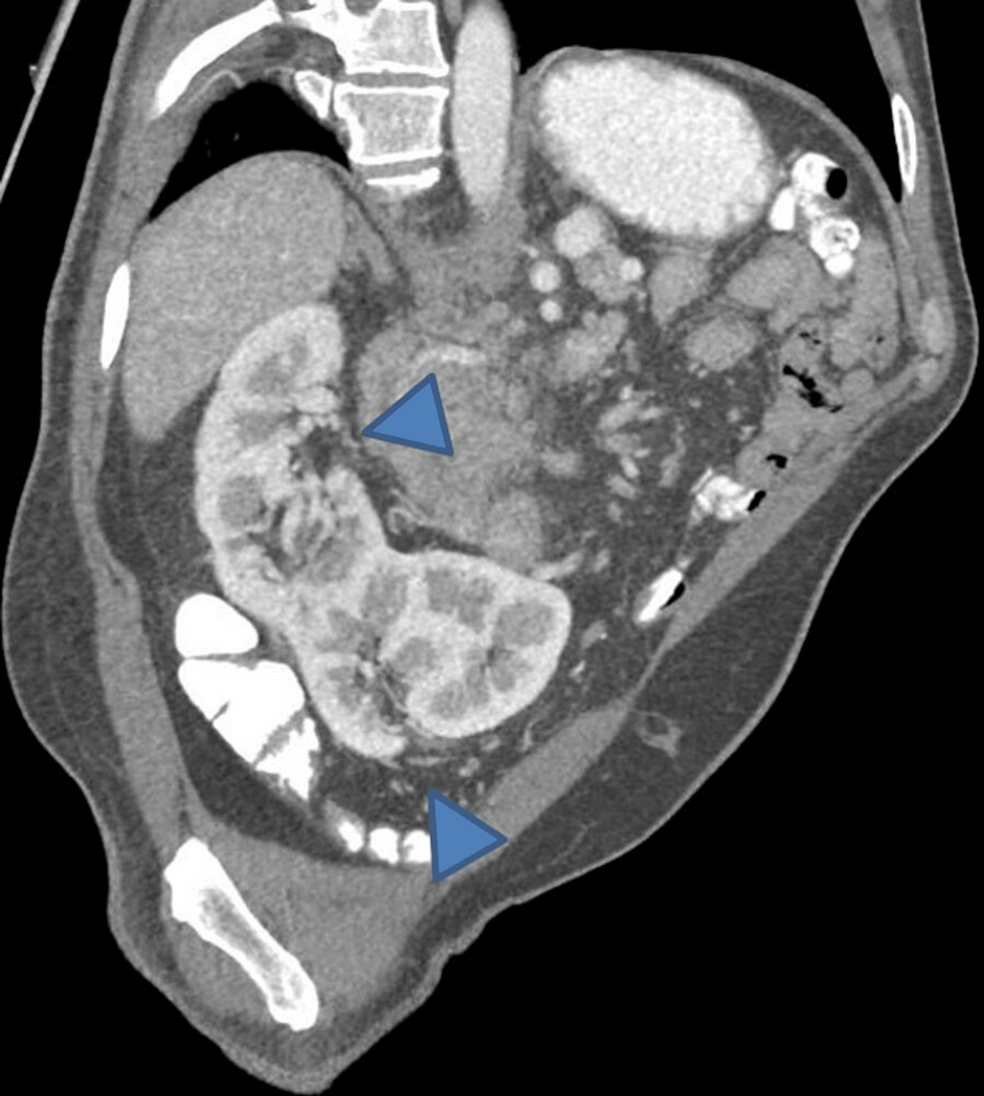
Curved multiplanar reconstruction image showing S-shaped kidney. The image was reconstructed along the axis of the kidney and based on the corticomedullary phase image. The presence of two distinct renal sinuses (arrowheads) with different orientations can be appreciated.

During the CT assessment, an incidental discovery of a left-to-right crossed renal fused ectopic kidney (S-shaped) with no associated hydronephrosis was made (Figure 2).

The calculated glomerular filtration rate (GFR) indicated mild impairment at 73 mL/min. To the best of our knowledge, this case represents only the third reported instance of crossed renal fused ectopia in a patient with prostatic cancer.

In the therapeutic approach for this patient, the decision to commence a tripartite treatment strategy was influenced by the high-volume nature of the disease and the patient's favorable performance status. The regimen comprised androgen deprivation therapy (ADT), supplemented with darolutamide, and the administration of docetaxel. Subsequent evaluation at a six-month interval demonstrated a positive therapeutic response, as indicated by a reduction in prostate-specific antigen (PSA) levels to 0.3 ng/ml and observable diminution in metastatic lesions.

## Discussion

The kidney undergoes intricate embryological development involving the reciprocal interaction between the mesonephric duct-derived ureteric bud and the metanephros, which represents the most caudal segment of the nephrogenic cord [1]. This developmental process commences in the early fourth week of gestation [2-4]. Subsequently, during the sixth to eighth week, notable transformations occur in the embryonic kidneys, involving their ascent from the pelvic region along the posterior abdominal wall to attain their designated anatomical position [6].

The aberrant positioning of the kidney on the opposite side of its normal location results from the abnormal migration of the ureteric bud and metanephric blastema across the midline [1]. Various theories have been postulated to elucidate the underlying mechanisms driving this phenomenon [7]. According to one hypothesis, the deviant placement of the umbilical artery influences the kidney's migration, prompting it to follow a path of minimal resistance and consequently relocate to the contralateral side [4]. An alternative theory proposes that the crossing over of the ureteric bud arises due to the excessive bending and rotation of the embryo's caudal extremity, impeding the fusion between the ureteric bud and the metanephric blastema [8]. Furthermore, there is evidence supporting the notion of contralateral metanephric blastema stimulation concurrent with regression of the metanephros on the ipsilateral side [8]. the presence of artery variations might lead to abnormal kidney development and changed positions and size of the kidneys [9].

The majority of cases involving crossed fused renal ectopia (CFRE) exhibit no symptoms and are typically identified incidentally, similar to our patient's case [4]. However, in instances where symptoms do arise, abdominal or flank pain, dysuria, recurrent urinary tract infections, and hematuria are most frequently reported [1-3]. Furthermore, CFRE may be accompanied by associated congenital malformations affecting the urogenital, gastrointestinal, and musculoskeletal systems. Among urogenital abnormalities, conditions such as ureterocele, kidney stones, blockage of the ureteropelvic junction, and, rarely, cancer have been observed [10, 11]. Vesicoureteral reflux (VUR) is the most common concurrent abnormality in CFRE cases [12, 13].

To establish a diagnosis of crossed-fused renal ectopia, various diagnostic methods such as abdominal ultrasonography, kidney-ureter-bladder X-ray (KUB), retrograde pyelography, and three-dimensional computed tomography (3D CT) have been employed before any intervention [14, 15]. Initially, ultrasonography is useful in identifying the absence of a kidney. Subsequently, KUB and retrograde pyelography aid in determining the size and location of any existing stones. It is crucial to carefully examine the findings obtained from 3D CT as they provide valuable information about essential blood vessels, allowing for safe access during surgery while minimizing the risk of bleeding and potential complications.

Vascular anatomy in crossed fused ectopic kidneys exhibits considerable variations, and the crossed ectopic kidney commonly displays diminished functional capacity [5]. Notably, in approximately 25% of cases with CFRE, the arterial blood supply originates from the superior abdominal aorta, while in the remaining 75%, the arterial supply arises from either the inferior abdominal aorta or the iliac arteries [6, 16]. In the case presented, the main left renal artery originates from the left iliac artery.

## Conclusions

The prognosis for individuals with CFRE is generally favorable in the absence of complications and additional associated malformations. Specific primary treatment approaches for CFRE are not available. However, management primarily focuses on addressing the associated pathologies, most commonly urolithiasis and VUR. It is crucial to have a comprehensive understanding of the aberrant anatomy before planning any surgical intervention in the renal region. Adequate imaging with angiography is strongly recommended before surgical procedures. The significance of this report lies in the underscored emphasis on the importance of employing precise imaging techniques and tailored management strategies for patients harboring such anatomical variations.
